# Malignant Gastrointestinal Neuroectodermal Tumor (clear Cell Sarcoma-like Tumor of The Gastrointestinal Tract) of The Small Intestine in a 12-year-old Boy

**DOI:** 10.34763/devperiodmed.20182204.358363

**Published:** 2019-01-14

**Authors:** Przemysław Wolak, Andrzej Wincewicz, Piotr Czauderna, Michał Spałek, Anna Kruczak, Sławka Urbaniak-Wąsik, Janusz Ryś, Elżbieta Michalak, Martyna Woltanowska, Stanisław Sulkowski

**Affiliations:** 1 Department of Pediatrics, Pediatric and Social Nursing, Institute of Nursing and Midwifery, Faculty of Medicine and Health Sciences, Jan Kochanowski University, Kielce, Poland; 2 Department of Pediatric Surgery, Urology and Traumatology, Voivodeship Specialist Hospital, Kielce, Poland; 3 Non-Public Health Care Unit, Department of Pathology, Kielce, Poland; 4 Department of Surgery and Urology for Children and Adolescents, Medical University of Gdańsk, Gdańsk, Poland; 5 Department of Anatomy, Faculty of Medicine and Health Sciences, Jan Kochanowski University, Kielce, Poland; 6 Department of Clinical Imaging, Holy Cross Center of Oncology, Kielce, Poland; 7 Department of Tumor Pathology, Center of Oncology, Maria Skłodowska-Curie Memorial Institute, Kraków, Poland; 8 Department of Pathomorphology, National Research Institute of Mother and Child, Warsaw, Poland; 9 NZOZ VITA Białystok, Poland; 10 Department of General Pathomorphology, Medical University of Białystok, Białystok, Poland

**Keywords:** chemotherapy, fluorescent in situ hybridization, gastrointestinal neuroectodermal tumor, ileus, thermal ablation, chemioterapia, fluorescencyjna hybrydyzacja in situ, guz neuroektodermalny przewodu pokarmowego, niedrożność jelita, termoablacja

## Abstract

The aim of this paper is a clinical and anatomopathological demonstration of a malignant lesion, a gastrointestinal neuroectodermal tumor (GNET), as an exceedingly rare cause of ileus in the pediatric population. Specifically, we present the case of a 12-year-old boy who showed dramatic weight loss, hypochromic anemia, fever, dehydration, exaggerated granulation of the terminal ileum, and mechanical ileus due to the obstruction by an intramural tumor of the small intestine. A 50cm-long part of the small intestine with pathological stricture was surgically removed, sampled and routinely fixed and stained with hematoxylin and eosin. The additional immunostains that were preformed were: PAS, S-100, HMB-45, NSE, LCA, CK AE1 / AE3, desmin, SMA, vimentin, CD99, NSE, synaptophysin, WT-1, calretinin, and DOG-1. Moreover, fluorescent in situ hybridization (FISH) with the EWSR1 Break Apart FISH Probe was applied. The neoplasm was composed of nests and alveolar patterns of frankly malignant clear cells with immunoreactivity to S-100, vimentin, and CD 99. The FISH technique detected chromosomal breaking at 22q12. The tumor metastasized to both the mesenteric lymph nodes and a number of hepatic segments. With several chemotherapy protocols, repeat laparotomies, and liver thermal ablations, the patient had a 1.5-year-long survival from the moment of diagnosis. The diagnosis of this malignancy requires both histopathological evaluation and molecular analysis, and the follow-up is based on careful clinical imaging of the neoplastic spread in order to apply proper surgical and oncological treatments. In conclusion, the clinical course of GNET was highly aggressive.

## Streszczenie

Celem artykułu jest prezentacja przebiegu klinicznego oraz sposobu ustalenia rozpoznania złośliwego guza neuroektodermalnego przewodu pokarmowego (GNET), będącego rzadką przyczyną niedrożności jelit u dzieci. Przedstawiamy przypadek 12-letniego chłopca, który wykazywał dramatyczny spadek masy ciała, niedokrwistość niedoborową, gorączkę, odwodnienie, wydatną granulację błony śluzowej końcowego odcinka jelita krętego w badaniu endoskopowym oraz niedrożność mechaniczną z powodu obecności śródściennego guza jelita cienkiego. Usunięty chirurgicznie 50-centymetrowy fragment jelita cienkiego z patologicznym zwężeniem utrwalono rutynowo, pobrano wycinki i wybarwiono hematoksyliną i eozyną. Dodatkowo wykonano panel barwień immunohistochemicznych: PAS, S-100, HMB-45, NSE, LCA, CK AE1/AE3, desmina, SMA, wimentyna, CD99, NSE, synaptofizyna, WT-1, kalretynina i DOG-1. Zastosowano też technikę fluorescencyjnej hybrydyzacji in situ (FISH) z użyciem sondy EWSR1 Break Apart FISH. Nowotwór był zbudowany z gniazd i układów alweolarnych złośliwych jasnych komórek o immunoreaktywności do S-100, wimentyny i CD 99. Dzięki FISH potwierdzono pęknięcia chromosomalne w 22q12. Guz dał przerzuty do krezkowych węzłów chłonnych i kilku segmentów wątroby. Dzięki wielokrotnym kursom chemioterapii, kolejnym laparotomiom i termoablacjom wątroby przeżycie pacjenta od momentu ustalenia diagnozy wyniosło 1,5 roku. Konkludując, przebieg kliniczny GNET był wysoce agresywny. Aktualnie ustalenie rozpoznania GNET wymaga nie tylko oceny histopatologicznej, ale i analizy molekularnej, a dokładne obrazowanie rozsiewu nowotworowego leży u podstaw wybrania właściwej terapii chirurgicznej i onkologicznej.

## Introduction

Small bowel obstruction in childhood can be caused by numerous lesions, such as intestinal intussusception, hernias, Crohn’s disease, Meckel’s diverticula, or developmental anomalies, not to mention remnants of the omphalomesenteric duct [1, 2]. Mechanical ileus can also be observed in the course of malignancies, of which the most numerous are lymphomas and other hematological malignancies [3, 4, 5, 6]. Sporadically, a ruptured Wilms tumor may give such a clinical outcome. In contrast, in adulthood, lymphomas are outnumbered by gastrointestinal stromal tumors, or GISTs, as the primary causes of small bowel obstruction [7]. Benign neoplasms constitute a rather minor fraction among the causes of bowel obstruction in pediatric patients, as evidenced by a case of lipoma that led to intestinal intussusceptions [8]. Moreover, hemangiomas have been described as factors triggering obstruction of the small intestine, and even perforations with internal bleeding and peritonitis, particularly in the jejunum [9].

The aim of the present paper is a clinical and anatomopathological demonstration of an unusual case of a young teen who developed mechanical ileus due to a malignant gastrointestinal neuroectodermal tumor (GNET; or a clear cell sarcoma-like tumor of the gastrointestinal tract) of the small intestine.

## Case study

**Fig. 1:**
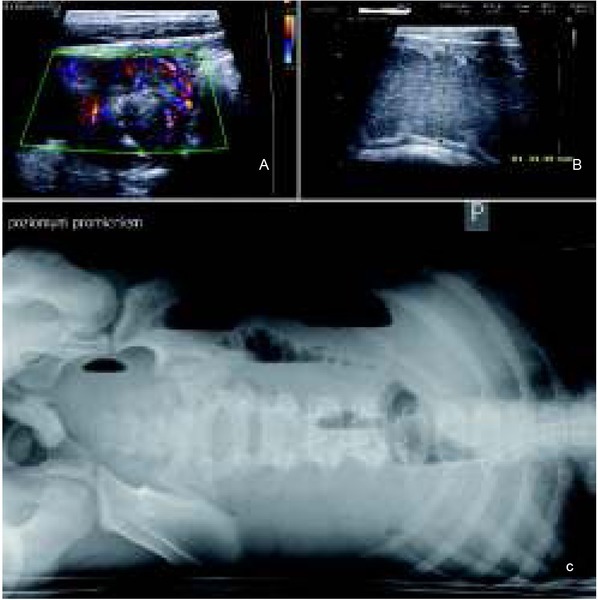
Imaging of the intramural tumor of the small intestine. A. Abdominal ultrasound before the onset of ileus, visible thickening of small intestine walls to 7 mm and increased vascularity in color Doppler study. B. Abdominal ultrasound at the time of malignant bowel obstruction - note a significant distention of the intestine. C. X-ray examination of the abdominal cavity in the supine position confirming bowel obstruction. Ryc. 1. Obrazowanie śródściennego guza jelita cienkiego. A. Obraz ultrasonograficzny jamy brzusznej przed wystąpieniem niedrożności jelit − wśród pętli jelitowych widoczne 7-milimetrowe zgrubienia ściany jelita cienkiego ze zwiększonym przepływem naczyniowym w badaniu kolorowym Dopplerem. B. Obraz ultrasonograficzny jamy brzusznej po wystąpieniu niedrożności przewodu pokarmowego - znaczne poszerzenie światła jelita cienkiego. C. Badanie rentgenowskie jamy brzusznej w pozycji leżącej - niedrożność jelit.

A 12-year-old boy was hospitalized for an intermittent midabdominal pain and the presence of free fluid in the hypogastrium. On ultrasound, his small intestine loops demonstrated enhanced vascularization (Fig. 1A), segmental narrowing of their lumen with apparent thickening of the intestinal wall up to 4-7mm (Fig. 1B), as well as distension by air and fluid as a hallmark of bowel obstruction (Fig. 1C). His mesenteric lymph nodes were enlarged measuring up to 13 mm in their longitudinal axis. Before admission, he had visibly lost weight: approximately 10 kg over a period of 2 months. In addition, hypochromic anemia, fever, and dehydration were present. Specimens collected at colonoscopy revealed edema of the ileocecal valve, erosions and hyperaemia of intestinal mucus, and ulcerations of the small intestine with pronounced inflammation. Therefore, a provisional suspicion of Leśniowski-Crohn’s disease was made. Since a 38°C fever, significant dehydration, green regurgitations, and anemia continued for the next 3 weeks in spite of symptomatic treatment, his general condition worsened. Explorative laparotomy was performed for life saving indications, and a tumor mass was found. A 50 cm-long segment of the small intestine with an abnormal stricture was removed and an enterocutaneous fistula created for the relief of malignant bowel obstruction.

**Fig. 2:**
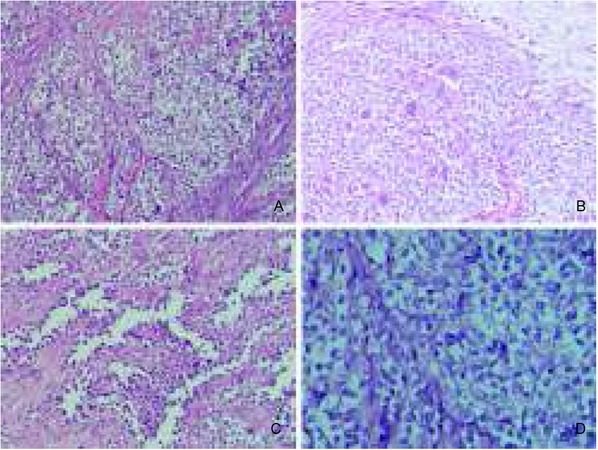
Histopathological appearance of malignant gastrointestinal neuroectodermal tumor (or, clear cell sarcoma-like tumor of gastrointestinal tract). A. Nested and sheet-like patterns of malignant cells (magnification: x200). B. Osteoclast-like multinucleated giant cells scattered in tumor fields (magnification: x 200). C. Alveolar and pseudopapillary patterns of tumor growth (magnification: x200). D. Elongated or epithelioid morphology of tumor cells together with evident clearing of cytoplasm (magnification: x 400). Ryc. 2. Obraz histopatologiczny złośliwego nowotworu neuroektodermalnego przewodu pokarmowego (tj. guza przewodu pokarmowego podobnego do mięsaka jasnokomórkowego). A. Pola i gniazda komórek złośliwych (powiększenie: x200). B. Olbrzymie komórki podobne do osteoklastów rozproszone w utkaniu guza (powiększenie: x200). C. Układy pęcherzykowe i pseudobrodawkowate w guzie (powiększenie: x200). D. Wydłużone i nabłonkowowate komórki nowotworowe z wyraźnym rozjaśnieniem cytoplazmy (powiększenie: x400).

**Fig. 3:**
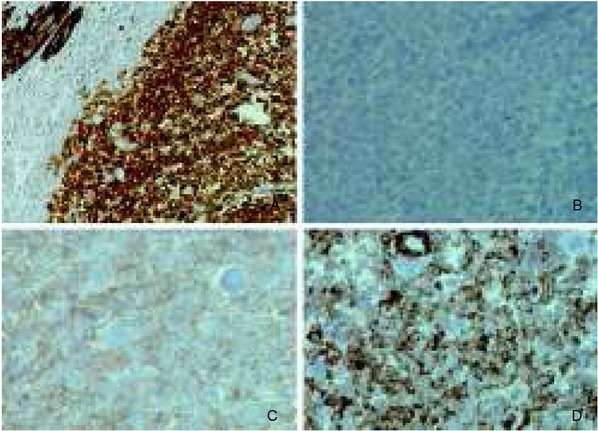
Immunoprofile of malignant gastrointestinal neuroectodermal tumor. A. Strong nuclear and cytoplasmic positivity to S-100 (magnification: x200). B. Negativity to HMB-45 (magnification: x400). C. Positivity to CD99 (magnification: x400). D. Positivity to vimentin (magnification:x400). Ryc. 3. Immunoprofil złośliwego guza neuroektodermalnego przewodu pokarmowego. A. Silnie dodatni immunohistochemiczny odczyn jądrowy i cytoplazmatyczny na S-100 (powiększenie: x200). B. Ujemny odczyn na HMB-45 (powiększenie: x400). C. Dodatni odczyn na CD99 (powiększenie: x400). D. Dodatni odczyn na wimentynę (powiększenie: x400).

The anatomopathological examination revealed a 4.0x3.0x1.5 cm intramural tumor with malignant cells arranged in nests and sheets (Fig. 2A) or in alveolar and pseudopapillary patterns (Fig. 2C). Osteoclast-like multinucleated giant cells populated the tumor in a scattered manner (Fig. 2B). The tumor cells were of elongated epithelioid shape and apparent malignant morphology (Fig. 2D). Additional immunostains were preformed: PAS, S-100, HMB-45, NSE, LCA, CK AE1 / AE3, desmin, SMA, vimentin, CD99, NSE, synaptophysin, WT-1, calretinin, and DOG-1. Furthermore, *fluorescent in situ hybridization* (FISH) was applied. The staining of S-100, CD99 and vimentin were strongly positive (Fig. 3A, C, and D), whereas there was less reactivity for PAS, NSE, WT-1, and LCA. Staining for HMB-45 was negative (Fig. 3B).

**Fig. 4:**
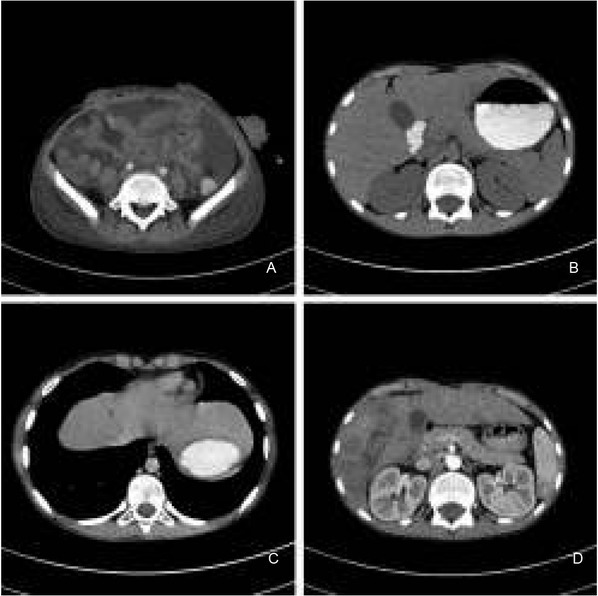
Further course of the disease as studied with computed tomography. A. Tomographic image of the abdomen after first surgery, large amounts of free fluid present. B. Abdominal tomography shortly after first surgery, no metastatic foci in the liver were detected. C. Abdominal tomography at four months after first surgery, note a small hypodense metastatic liver lesion. D. Computed tomography of the abdomen at a later time, numerous liver metastases. Ryc. 4. Dalszy przebieg choroby w obrazowaniu tomografią komputerową. A. Tomografia brzucha po pierwszej operacji - znaczna ilość wolnego płynu w jamie brzusznej. B. Tomografia brzucha bezpośrednio po operacji, z uwidocznieniem wątroby − bez dostrzegalnych ognisk przerzutów. C. Jama brzuszna w cztery miesiące po operacji - ognisko przerzutowe o niewielkiej hipodensyjności w środku wątroby. D. Jama brzuszna w późniejszym terminie - kolejne liczne przerzuty do wątroby.

In the anatomopathological differential diagnosis, the following entities were included: alveolar rhabdomyosarcoma, Ewing’s sarcoma/primitive neuroectodermal tumor, GIST, and synovial sarcoma. The definite pathological diagnosis was specified as: clear cell sarcoma-like tumor of the gastrointestinal tract (CCSL-GT), finally designated with its most recent and updated nomenclature: Malignant Gastrointestinal Neuroectodermal Tumor. This diagnosis was additionally confirmed at molecular level. Namely, the EWSR1 Break Apart FISH Probe detected chromosomal breakage at 22q12. A postoperative computed tomography (CT) scan revealed complete atelectasis in the inferior segments of the left lung, hydrothorax, and the persistence of free fluid retention around the urinary bladder and in both iliac fossae. To the right of the aortic bifurcation, another irregular fluid-filled space (33.0x28.0x35.0 mm) was found and interpreted as packages of mesenteric lymph nodes with substantial dissolution of their structures (Fig. 4A). Abdominal tomography shortly after the first surgery revealed there were no detectable metastatic foci in the liver (Fig. 4B). On postoperative Day 11, a second surgery was done: the mesenteric polycyclical mass was surgically removed, the intestine reunited with end-to-end anastomosis, and omentectomy performed. Among the 11 examined lymph nodes from the polycyclical mass, there were 4 nodal metastazes, with the largest metastatic lesion 1.7-cm in diameter and containing a 0.9-cm focus of necrosis. The patient was given CWS 2006 VAIA III chemotherapy (vincristine, adriamycin (doxorubicin), afosfamide, dactinomycin), and later CEVAIE (carboplatin, epirubicin, vincristine, actinomycin D, ifosfamide and etoposide). However, 8 weeks from the first laparotomy, repeated CT and magnetic resonance imaging (MRI) showed 8-16 mm, large metastases to several liver segments (Fig. 4C and D). At 6 months from diagnosis, these lesions were biopsied and histopathologically confirmed to be of metastatic nature. Consequently, thermal ablation of the detected lesions was the next procedure. During the third laparotomy, a mesenteric tumor mass and a tumor bordering both the right kidney and liver were removed. Although CEVAIE chemotherapy was continued, unfortunately, new lesions in the liver emerged. Therefore, etoposide was added to the therapy, yet the patient suffered substantial marrow aplasia after each course of chemotherapy, with fever as a constant symptom. The second thermal ablation of liver foci followed. Due to the progression of liver metastases, pazopanib (a multi tyrosine kinase inhibitor) was introduced. In spite of that, a subsequent abdominal cavity MRI revealed the presence of new hepatic lesions of metastatic appearance. Six weeks later, he was admitted to the hospital for the presence of free abdominal fluid accumulating up to the epigastric region. At that stage, fever up to 39°C, dyspnea related to physical activity, severe anemia, and leukocytosis were present. *Escherichia coli* was cultured from the aspirated abdominal fluid and broad-spectrum antibiotics were introduced. At 16 months from diagnosis, CT scans demonstrated neoplastic spread outside the liver and enlarged mediastinal and inguinal lymph nodes. Subject to careful palliative care at home, the patient died at 1.5 years of survival from diagnosis.

## Discussion

In the case presented, transabdominal ultrasound was the simplest and easiest method to confirm a clinically suspicious small bowel stricture, in line with a large study by Nakano et al., where intestinal strictures were classified into four distinct types [10]. Furthermore, our patient underwent surgical resection of stricture and end-to-end anastomosis, similarly as it had been done in a child with primary gastrointestinal B-cell high-grade B-cell Non-Hodgkin lymphoma who presented with a jejunal stricture related to chemotherapy-induced mucositis [11]. The tumor evoked symptoms largely shared with intestinal leiomyosarcoma with ileus [12]. Namely, our patient’s anemia was related to blood loss from repeated internal bleeding which is much more characteristic of invasive neoplasms than benign lesions of expansive growth [12]. Sadly, the patient followed a classical pattern of the quick spread of a lethal disease with intestinal obstruction, liver metastases, and very poor prognosis could be observed [13]. Typical locations of GNET are the small bowel, stomach, and colon [14]. When the small intestine is involved, the clinical picture usually comprises colic abdominal pain and vomiting as prominent symptoms [14], as was the case of our patient. Classically, the invaded intestinal wall is thickened and narrowed in some segment(s), with X-ray or CT radiological evidence at hand [14]. The management of and outcome in the boy described was similar to other reported cases of GNET [13, 14, 15].

Of note, clear cell sarcomas share a distinctive histopathological and molecular profile regardless of their location [15]. In 2003, Zambrano et al. were the first to report on 6 cases of GNET that were composed of S100-positive clear cell texture with scattered osteoclast-like multinucleated giant cells showing no reactivity to CD117 and melanocytic markers [16]. These authors defined morphological particularities of clear cell sarcoma-like tumors of the gastrointestinal tract as completely distinct entities. Subsequently, Friedrichs et al. pointed out the following differing features of a small bowel tumor in a 41-year-old patient: mixed alveolar and solid histological architecture, osteoclast-like giant cells present, coexpression of S-100 and vimentin, complete lack of HMB-45, melan-A, SMA, KIT receptor, desmin, CD-34 expression, and harbouring the following translocation: t(12;22)(q13;q12) [17]. Such a precise characterization was sufficient to constitute a new entity in pathology and it was called a clear cell sarcoma-like tumor of the gastrointestinal tract (CCSLT-GT). Later it was renamed GNET [14, 16]. The *EWSR1*-related anomaly is regarded as a genetic fingerprint of this entity [14]. However, this fingerprint is far from being a unique identifier of CSSLT-GT [18]. Actually, the EWSR1 gene presents with a promiscuous nature that makes this gene fuse with a multitude of partner genes resulting in a wide range of phenotypically similar or morphologically completely different tumors [18, 19]. Nonetheless, it is a widely spread and well acknowledged standard to combine molecular EWSR1 identification and pathological diagnosis of CCSLT-GT in each case. It is so because of its morphological similarity to malignant melanoma. CSSLT-GT resembles clear cell sarcoma that was historically designated as malignant melanoma of soft tissues due to its resemblance to malignant melanoma at the basic morphological level, as well as a similar immunoprofile and ultrastructure. CCSLT-GT shares its immunoprofile with malignant melanoma in case of S100 positivity but completely lacks HMB-45 and melan A immunoreactivity [14]. Importantly, clear secretory vesicles, dense core granules, occasional gap junctions, and lack of melanogenesis are ultrastructural traits of primitive neuroectodermal cells [14]. Therefore, the current updated designation of CCSLT-GT is: (Malignant) Gastrointestinal Neuroectodermal Tumor (GNET) in order to emphasize the assumed origin of this malignancy from gastrointestinal precursor cells of neuroectodermal nature that are unable to follow the melanocytic pathway of differentiation. This explains the hallmark positivity for strong neural markers: S-100, SOX10, CD56, synaptophysin, NB84, and NSE, and − to a lesser extent − for neurofilament protein [14]. In line, expression of SOX10 transcription factor is the evidence of schwannian or melanocytic differentiation and again justifies the tumor’s recent designation [20, 21]. In conclusion, in our diagnosis, in accordance with numerous records, we favor the more precise term GNET over an earlier synonym CCSLT-GT, to describe this rare anatomopathological entity [14, 16, 21, 22].
